# *TAC1b* mutation in *Candida auris* decreases manogepix susceptibility owing to increased *CDR1* expression

**DOI:** 10.1128/aac.01508-24

**Published:** 2024-12-18

**Authors:** Tatsuro Hirayama, Taiga Miyazaki, Rina Tanaka, Natsume Kitahori, Masataka Yoshida, Kazuaki Takeda, Shotaro Ide, Naoki Iwanaga, Masato Tashiro, Takahiro Takazono, Koichi Izumikawa, Katsunori Yanagihara, Koichi Makimura, Kazuhiro Tsukamoto, Hiroshi Mukae

**Affiliations:** 1 Department of Pharmacotherapeutics, Nagasaki University Graduate School of Biomedical Sciences 57927 , Nagasaki, Japan; 2 Department of Respiratory Medicine, Nagasaki University Hospital 57927 , Nagasaki, Japan; 3 Division of Respirology, Rheumatology, Infectious Diseases, and Neurology, Department of Internal Medicine, Faculty of Medicine, University of Miyazaki 12952 , Miyazaki, Japan; 4 Infectious Diseases Experts Training Center, Nagasaki University Hospital 57927 , Nagasaki, Japan; 5 Department of Infectious Diseases, Nagasaki University Graduate School of Biomedical Sciences 57927 , Nagasaki, Japan; 6 Department of Laboratory Medicine, Nagasaki University Hospital 57927 , Nagasaki, Japan; 7 Teikyo University Institute of Medical Mycology, Teikyo University 13094 , Tokyo, Japan; University of Iowa, Iowa City, Iowa, USA

**Keywords:** *Candida auris*, manogepix, *TAC1b*, *CDR1*, antifungal resistance, efflux pumps, CRISPR-Cas9

## Abstract

*Candida auris* is an emerging pathogenic fungus that is highly resistant to existing antifungal drugs. Manogepix is a novel antifungal agent that exerts antifungal activity by inhibiting glycosylphosphatidylinositol anchor biosynthesis. Although the mechanisms of resistance of *Candida* species to manogepix have been reported previously, those of *C. auris* are yet to be studied. To investigate the resistance mechanisms of *C. auris*, we exposed a clinical isolate (clade I) to manogepix *in vitro* and generated strains with reduced susceptibility to manogepix. A search for gain-of-function mutations that upregulate efflux pump expression confirmed the presence of the D865N amino acid mutation in *TAC1b*. We used the clustered regularly interspaced short palindromic repeats-Cas9 system to create a recovery strain (N865D) in which only this single nucleotide mutation was returned to the wild-type sequence. We generated a mutant strain by introducing only the D865N mutation into the parent strain and a different clade strain (clade III). The D865N mutant strains were clearly less susceptible to manogepix than the parental strains and exhibited high *CDR1* expression. Moreover, we generated a strain deficient in *CDR1* and confirmed that this strain had significantly increased susceptibility to manogepix. Thus, the present study demonstrated that the *TAC1b* mutation in *C. auris* upregulates *CDR1* expression and decreases its susceptibility to manogepix.

## INTRODUCTION

*Candida auris* is a multidrug-resistant yeast that causes invasive fungal diseases and healthcare-associated outbreaks worldwide. It is listed in the Urgent Threat group in “Antibiotic Resistance Threats in the US, 2019” by the Centers for Disease Control and Prevention (CDC) and in the Critical Priority group in “WHO fungal priority pathogen List, 2022.” Based on tentative breakpoints suggested by the CDC, approximately 90%, 30%, and <5% of isolates in the USA are resistant to fluconazole, amphotericin B, and echinocandins, respectively (1). Therefore, the development of new antifungal agents effective against *C. auris* is critical.

Manogepix is the active component of fosmanogepix, a new drug candidate currently in clinical trials for the treatment of invasive fungal infections (2, 3). A new class of antifungal agents exerts antifungal effects by inhibiting Gwt1, an enzyme essential for the maturation and anchoring of fungal glycosylphosphatidylinositol (GPI)-anchored mannoproteins to the cell membrane (4, 5). Some fungal adhesion and virulence factors are derived from GPI-anchored proteins, and the inhibition of Gwt1 can reduce hyphal formation and biofilm formation, as well as malformation of cell morphology (6). Because manogepix does not inhibit PIGW, the mammalian ortholog of Gwt1, it is considered to selectively act on fungi (4, 6). Fosmanogepix exhibits broad-spectrum antifungal activity against *Candida* species, including *C. auris*, *Aspergillus,* and *Cryptococcus* species, and other major pathogenic fungi (2, 7–11). These characteristics make it a promising antifungal drug for clinical application, and a phase III clinical trial is currently underway for candidemia and invasive candidiasis (NCT05421858).

Although amino acid mutations in *GWT1* (12) and high expression of efflux pumps (13) have been identified as mechanisms underlying the resistance of *Candida* spp. to manogepix, the mechanism of resistance in *C. auris* has not been reported. In this study, we aimed to evaluate the possible development of manogepix resistance in *C. auris* and to analyze the mechanism of reduced susceptibility.

## RESULTS

### *In vitro* susceptibility and determination of *TAC1b* mutations in manogepix-exposed isolates and mutation analysis

*C. auris* NCPF8985 (clade I; South Asia clade) and *C. auris* NCPF8977 (clade III; South Africa clade) (14) were used in this study. The minimum inhibitory concentration (MIC) of manogepix for both strains was 0.03 µg/mL. NCPF8985 was applied to yeast extract-peptone-dextrose (YPD) agar containing manogepix, and the MICs of the 100 isolated strains are shown in Table S1. The highest recorded MIC was 0.5 µg/mL, and four isolates achieved this result. After repeated subculture of these four strains in YPD broth devoid of manogepix, the MICs of the two strains decreased to 0.125 µg/mL, whereas that of the other two strains remained 0.5 µg/mL. These two strains, denoted as R2 and R38, were sequenced for *GWT1* and efflux pump-related genes. Direct sequencing revealed that both strains possessed a single nucleotide mutation (G2593A) in *TAC1b* that transformed the 865th aspartic acid to asparagine (D865N). The analysis did not reveal any amino acid mutations in *GWT1* or other efflux pump-related genes.

### D865N amino acid mutation in *TAC1b* reduced the susceptibility of *C. auris* to manogepix

**Fig 1:**
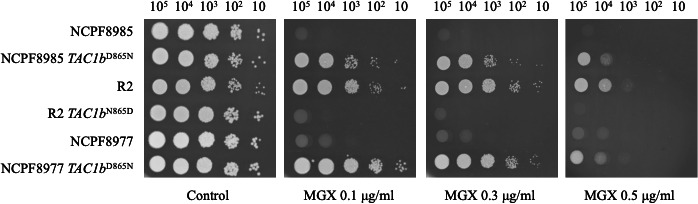
Mutation in *TAC1b* reduced manogepix susceptibility in *C. auris*. The effect of D865N mutation in *TAC1b* on manogepix susceptibility was examined using a spot dilution assay in the wild-type and *TAC1b*^D865N^ mutants. Log-phase cells were serially diluted and spotted onto SC agar plates containing a gradient of manogepix. The plates were then incubated at 30°C for 48 h.

To investigate the effect of the D865N amino acid mutation in *TAC1b* in *C. auris* on manogepix susceptibility, we introduced the mutation (G2593A) in the wild-type strain NCPF8985 and reversed the mutation (A2593G) in the mutant strain R2 using the CRISPR-Cas9 method. Furthermore, we introduced the D865N mutation in *TAC1b* into the NCPF8977 clade. The introduction of D865N into *TAC1b* increased the manogepix MIC from 0.0 to 0.25 μg/mL in both NCPF8985 and NCPF8977. Additionally, reverting the amino acid mutation in D865N reduced the manogepix MIC of R2 from 0.5 to 0.06 μg/mL (Table 1). The spot dilution assay revealed reduced susceptibility of *TAC1b*^D865N^ strains to manogepix (Fig. 1).

**TABLE 1:** Manogepix MICs for the strains used in this study

Strain	Description	Manogepix MIC (μg/mL)
NCPF8985	Parental strain	0.03
NCPF8985 *TAC1b*^D865N^	NCPF8985 *TAC1b*^D865N^::*NatR*	0.25
R2	NCPF8985 evolved strain (*TAC1b*^D865N^)	0.5
R38	NCPF8985 evolved strain (*TAC1b*^D865N^)	0.5
R2 *TAC1b*^N865D^	R2 *TAC1b*^N865D^::*NatR*	0.06
NCPF8977	Parental strain	0.03
NCPF8977 *TAC1b*^D865N^	NCPF8977 *TAC1b*^D865N^::*NatR*	0.25
NCPF8985 *tac1b*Δ	NCPF8985 *tac1b*Δ::*NatR*	0.03
NCPF8985 *cdr1*Δ	NCPF8985 *cdr1*Δ::*NatR*	0.016
R2 *tac1b*Δ	R2 *tac1b*Δ::*NatR*	0.06
R2 *cdr1*Δ	R2 *cdr1*Δ::*NatR*	0.06

### D865N amino acid mutation in *TAC1b* increased *CDR1* expression

**Fig 2:**
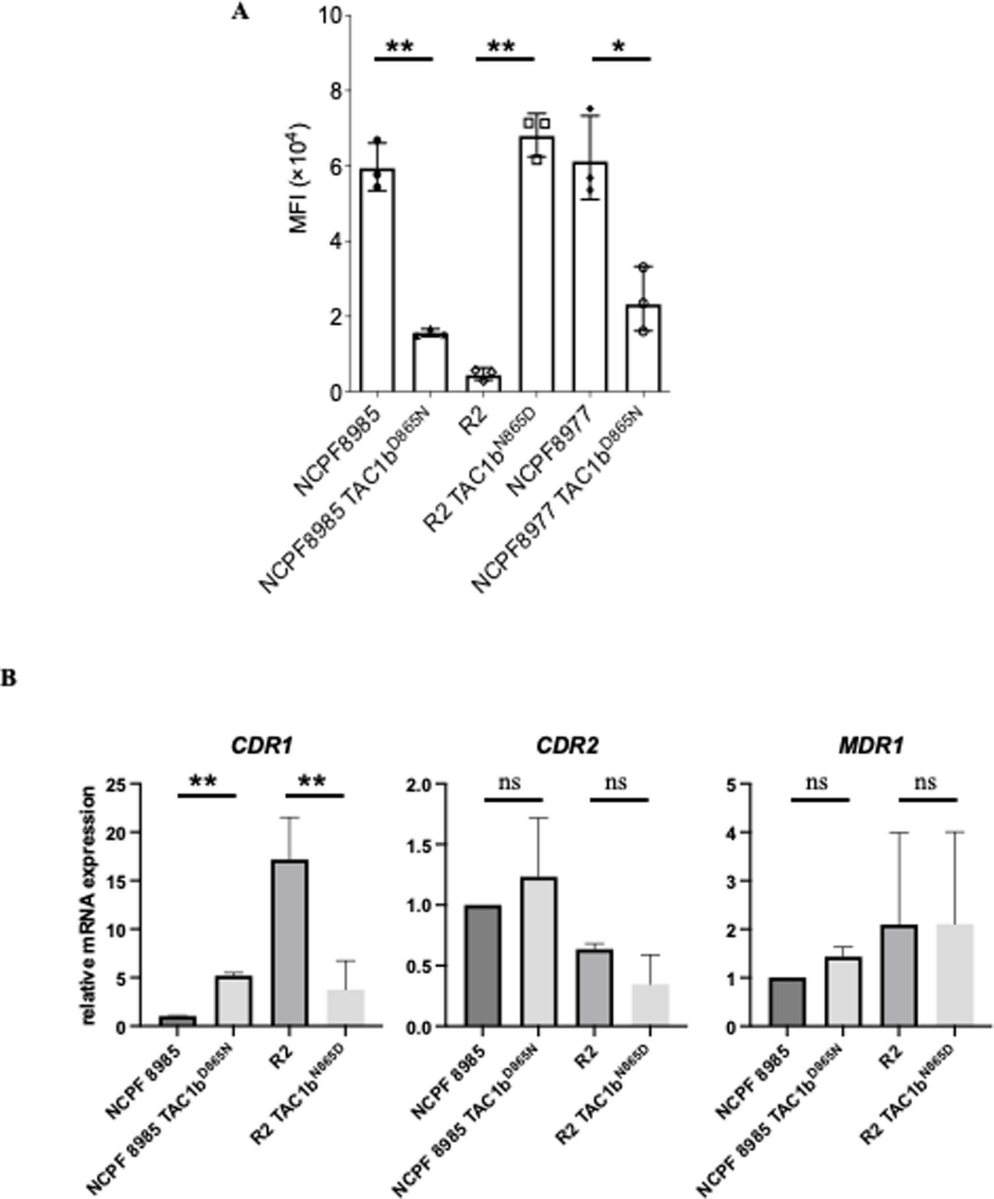
Mutation in *TAC1b* increased the expression level of *CDR1* and activated the efflux pump. (**A**) Intracellular concentration of the fluorescent dye Nile red, a substrate of the efflux pump, was measured through flow cytometry. Nile red accumulation was reduced in the *TAC1b* D865N mutants. Median fluorescence intensity (MFI) was measured in three independent experiments. The mean ± standard error is shown. Statistical analysis was performed using two-tailed Student’s *t*-test. *P < 0.05, **P < 0.01. (**B**) Real-time qRT-PCR was performed to examine the expression of efflux pump-related genes. Gene expression level of NCPF8985 was defined as one in each assay and was normalized to that of *ACT1*. The expression levels of *CDR1* were upregulated in *TAC1b* D865N mutants with decreased susceptibility to manogepix. The mean ± standard error of three independent experiments is shown.

*TAC1b* is a putative transcriptional activator of the drug-responsive transporter genes *CDR1* and *CDR2*, which are associated with fluconazole resistance (15). To examine whether the reduced susceptibility to manogepix was due to increased efflux pump activity, intracellular concentrations of the fluorescent dye Nile red, which accumulates within lipid membranes and is actively expelled from cells through efflux mechanisms (16), were measured using flow cytometry. Both NCPF8985 and NCPF8977 strains showed reduced accumulation of Nile red compared to the parental strain after the introduction of the *TAC1b* D865N mutation. In contrast, reversing the *TAC1b* mutation (N865D) in the R2 strain increased Nile red accumulation compared to that in the parental strain (Fig. 2A; Fig. S1). Real-time quantitative reverse transcription PCR (qRT-PCR) was performed to examine the expression of genes related to efflux pumps, including *CDR1*, *CDR2,* and *MDR1. CDR1* expression was approximately fivefold higher in the *TAC1b*^D865N^ strain than in the wild-type strain, and similarly, its expression was approximately fivefold lower in the R2 *TAC1b*^N865D^ strain than in the R2 strain. The *CDR2* and *MDR1* expression levels did not change significantly (Fig. 2B). The results indicated that the *TAC1b* mutation in *C. auris* upregulated *CDR1* expression and activated efflux pumps.

### Loss of Cdr1 or Tac1b increased manogepix susceptibility in the *TAC1b* mutant

**Fig 3:**
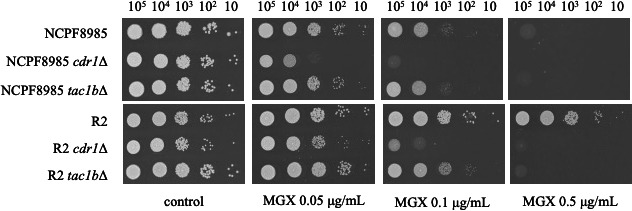
Loss of Cdr1 or Tac1b restored manogepix susceptibility in the *TAC1b* mutant strain. The effect of deletion of *CDR1* or *TAC1b* on manogepix susceptibility was examined using a spot dilution assay in the wild-type and *TAC1b*^D865N^ mutant. Log-phase cells were serially diluted and spotted onto SC agar plates containing a gradient of manogepix. The plates were then incubated at 30°C for 48 h.

To further evaluate the effects of *CDR1* and *TAC1b* on manogepix susceptibility, we deleted *CDR1* and *TAC1b* in the wild-type strain NCPF8985 and the *TAC1b*-mutant strain R2 using the CRISPR-Cas9 method. Deletion of *CDR1* reduced the MIC of the wild-type strain only slightly, from 0.03 to 0.016 μg/mL, but reduced that of the mutant strain greatly, from 0.5 to 0.06 μg/mL. Similarly, although deletion of *TAC1b* did not affect the MIC of the wild-type strain, it did reduce the MIC of the mutant strain from 0.5 to 0.06 μg/mL (Table 1). The effects of *CDR1* and *TAC1b* loss on susceptibility were further confirmed using spot dilution assays (Fig. 3). Taken together, these results indicate that activation of the manogepix efflux pump by the Tac1b-Cdr1 pathway is the primary mechanism underlying reduced manogepix susceptibility in *TAC1b* mutants.

**Fig 4:**
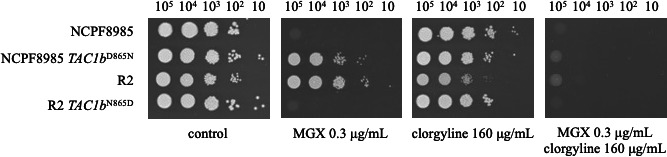
Combination of efflux pump inhibitors restored manogepix susceptibility in *TAC1b*-mutant strains. The effect of clorgyline, an efflux pump inhibitor, in combination with manogepix was examined using a spot dilution assay in wild-type and *TAC1b*^D865N^ mutants. Log-phase cells were serially diluted and spotted onto SC agar plates containing the indicated concentrations of manogepix and clorgyline. The plates were then incubated at 30°C for 48 h.

Furthermore, a spot dilution assay was performed using clorgyline, an inhibitor of the ATP-binding cassette (ABC) transporter (17), to confirm the combined effect of efflux pump inhibitors on low-manogepix-susceptibility strains. As expected, the combination of clorgyline and manogepix increased manogepix susceptibility in *TAC1b* mutants (Fig. 4).

### D865N amino acid mutation in *TAC1b* reduced the susceptibility of *C. auris* to fluconazole

**Fig 5:**
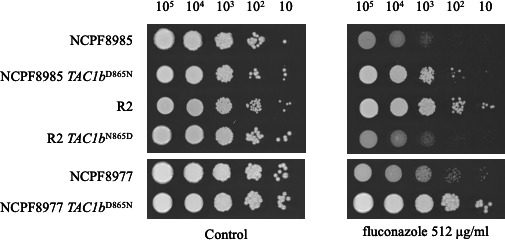
Mutation in *TAC1b* reduced fluconazole susceptibility in *C. auris*. The effect of D865N mutation in *TAC1b* on manogepix susceptibility was examined using a spot dilution assay in the wild-type and *TAC1b*^D865N^ mutants. Log-phase cells were serially diluted and spotted onto SC agar plates containing 512-µg/mL fluconazole. The plates were then incubated at 30°C for 48 h.

A spot dilution assay was performed to determine whether the D865N amino acid mutation in *TAC1b* also affects fluconazole susceptibility. The results showed that the *TAC1b*^D865N^ strains were less susceptible to fluconazole (Fig. 5). The MICs of fluconazole for the parental strains NCPF8985 and NCPF8977 exceeded 256 µg/mL, making it impossible to assess susceptibility using broth microdilution methods.

## DISCUSSION

In this study, we analyzed the mechanism underlying reduced manogepix susceptibility in *C. auris*. Mutations in *GWT1*, the target gene of manogepix, were previously reported to reduce the susceptibility to manogepix in *Candida albicans*, *Candida glabrata*, and *Candida parapsilosis* (12); however, in this study, no *GWT1* mutation was found in low-susceptibility strains of *C. auris*. Mechanisms underlying reduced manogepix susceptibility, other than *GWT1* mutations, have already been reported, including activation of the ABC transporter genes *CDR11* and *SNQ2* via a gain-of-function mutation in the transcription factor *ZCF29* in *C. albicans* and activation of the major facilitator superfamily transporter gene *MDR1* in *C. parapsilosis* (13). Our study suggests that in *C. auris*, a gain-of-function mutation in the transcription factor *TAC1b* activates the ABC transporter Cdr1, thereby reducing manogepix susceptibility.

Previous reports have shown that gain-of-function mutations in *TAC1* cause azole resistance in *Candida* spp. by overexpressing the drug efflux pump Cdr1 (18, 19), and in *C. auris*, gain-of-function mutations in *TAC1b* cause Cdr1 and Cdr2 overexpression, resulting in azole resistance (20–22). Carolus et al. (20) reported that exposure of *C. auris* to fluconazole introduced a gain-of-function mutation in *TAC1b*, resulting in a 32-fold increase in fluconazole MIC. In this study, we found that manogepix exposure introduced a gain-of-function mutation in *TAC1b*, resulting in a 16-fold increase in manogepix MIC. *CDR1* expression remarkably increased in the *TAC1b*^D865N^ mutant strain, whereas *CDR2* expression did not. *CDR1* deletion remarkably increased manogepix susceptibility, suggesting that Cdr1 is involved in manogepix efflux. In addition, the combination of manogepix and efflux pump inhibitors restored manogepix susceptibility in *TAC1b*-mutant strains. According to the domain diagram of *TAC1b* presented by Rybak et al. (21), the *TAC1b* D865N mutation is located in the carboxy-terminal activation domain. A known mutation in this region is the frameshift mutation F862_N866del, which confers high resistance to fluconazole. Mutations in this region are likely to contribute significantly to the increased expression of *CDR1*.

More than 90% of *C. auris* isolates are resistant to fluconazole (23). The main mechanism for acquiring resistance is thought to be mutation or overexpression of *ERG11*, which encodes lanosterol 14-α-demethylase (24, 25), as well as activation of the efflux pumps Cdr1 and Mdr1 (26). Although manogepix showed excellent *in vitro* activity against *C. auris* clinical isolates (27, 28), it is important to note that fluconazole-resistant strains with elevated *CDR1* expression may be less susceptible to manogepix. In this study, we found that the *TAC1b*^D865N^ mutation, which occurred after exposure to manogepix, decreased susceptibility to manogepix and fluconazole, suggesting that *TAC1b* mutations, which have been reported to reduce susceptibility to fluconazole may also reduce susceptibility to manogepix. Indeed, a high correlation between fluconazole and manogepix MICs has been reported in *C. auris* and other *Candida* isolates (7, 29), and our current study suggested that the cause of this correlation may be activation of the efflux pump.

Our study has two limitations. First, the etiology of low manogepix susceptibility in the R2 strain isolated after manogepix exposure may extend beyond the *TAC1b* mutation, and rectification of the *TAC1b* mutation in R2 did not restore it to the MIC of the parental strain. When comparing the *cdr1*-deficient R2 strain with the *cdr1*-deficient parental strain, the R2 *cdr1*Δ mutant demonstrated less susceptibility. In addition, R2 had higher *CDR1* expression than the *TAC1b*^D865N^ mutant strain, and we were unable to overlook the possibility of there being factors other than *TAC1b* that regulate the expression of *CDR1*. Although the *TAC1b* mutation undoubtedly contributed significantly to the low manogepix susceptibility in R2, mechanisms other than *TAC1b* mutation may still exist for reduced susceptibility, and further investigation is warranted to elucidate this phenomenon. Second, the *in vivo* therapeutic effects of manogepix have yet to be verified. Whether the MIC of manogepix, which increased to 0.5 µg/mL in *TAC1b*-mutant strains, could result in therapeutic resistance *in vivo* or poor therapeutic efficacy in clinical practice remains to be confirmed. Future research is needed to verify whether *TAC1b*-mutant strains are resistant to manogepix treatment *in vivo*.

In conclusion, we demonstrated that the *TAC1b* mutation activates Cdr1 expression and reduces the susceptibility of *C. auris* to manogepix. Manogepix is a promising treatment for multidrug-resistant *C. auris* and is expected to be widely used in the future. Understanding resistance to manogepix in advance and taking countermeasures against it are important in this regard.

## MATERIALS AND METHODS

### *Candida* strains, culture conditions, and reagents

*C. auris* NCPF8985 (clade I; South Asia clade) and *C. auris* NCPF8977 (clade III; East Asia clade) (14) were used in this study. *C. auris* cells were propagated in YPD medium (1% yeast extract, 2% peptone, and 2% dextrose; Difco Laboratories, Detroit, MI, USA) or synthetic complete (SC) medium at 30°C. SC medium containing non-fermentable carbon sources (3% glycerol and 2% ethanol) instead of dextrose was used to examine the petite growth phenotype. Manogepix (Selleck Chemicals, Houston, TX, USA), fluconazole (Sigma-Aldrich, St. Louis, MO, USA) and clorgyline (Sigma-Aldrich, St. Louis, MO, USA) were dissolved in dimethyl sulfoxide and stored at –20°C until use.

### Drug susceptibility testing

The manogepix MICs were determined using broth microdilution according to CLSI M27-E4 and were defined as the concentration at 50% growth inhibition (30, 31). Spot dilution assays were performed as previously described (32). Briefly, log-phase cells grown in SC broth were collected and adjusted to a concentration of 2 × 10^7^ cells/mL. Serial 10-fold dilutions were prepared, and 5 µL of each dilution was spotted onto SC agar plates containing the desired concentration of the test compound. The plates were then incubated at 30°C for 48 h.

### *In vitro* exposure to manogepix to develop strains with reduced susceptibility to manogepix and sequence analysis of the generated isolates

Experiments were conducted to develop manogepix -insensitive strains in accordance with a previous report (12). YPD agar (150 mL) containing manogepix at a 4 × MIC was prepared in a 245- × 245-mm assay dish. An overnight culture of *C. auris* NCPF8985 in YPD broth was diluted to approximately 1 × 10^8^/mL. Subsequently, 1 mL of this diluted culture was spread onto YPD plates containing manogepix and incubated at 30°C for 3 days. Subsequently, 100 colonies that had grown were selected, and the MIC was determined.

Two isolates with the highest manogepix MIC (0.5 µg/mL) were selected for sequencing of *GWT1* and transcription genes that activate efflux pump (*TAC1a*, *TAC1b*, *MRR1*, and *ZCF29*). The primers used for amplification and sequencing are listed in Table S2.

### Generation of *C. auris* mutants

We applied the CRISPR-Cas9 method (33, 34) to generate single nucleotide variants and gene deletion strains of *C. auris*. A diagram of this process is presented in Fig. S2.

To replace the single nucleotide mutation in *TAC1b*, a repair cassette featuring a nourseothricin-resistant (NatR) marker was obtained through fusion PCR using three DNA fragments with overlapping sequences. The *C. auris TAC1b* alleles of NCPF8985 (*TAC1b*^WT^) and R2 (*TAC1b*^D865N^) were amplified from genomic DNA. The construct was joined to a PCR product containing the NatR marker from the plasmid pNAT (35) and another with the downstream region of *TAC1b*, as depicted in Fig. S2A. To generate *CDR1* and *TAC1b* deletion strains, a fusion PCR approach was used, which involved combining three PCR products with overlapping sequences to obtain constructs containing the upstream and downstream flanking regions of *CDR1* or *TAC1b*, along with the NatR cassette, as depicted in Fig. S2B.

Transformations in *C. auris* were performed using the CRISPR-Cas9 method, which involved the use of genetic constructs described above. Ribonucleoprotein complexes were reconstructed using purified Cas9 and CRISPR RNAs (crRNAs). Using the Alt-R CRISPR HDR Design Tool (Integrated DNA Technologies, Coralville, IA, USA), the crRNAs were engineered to incorporate 20-bp sequences homologous to the regions flanking the Cas9 target site, both upstream and downstream (Fig. S2C and D). A mixture of crRNAs, Cas9 endonuclease (Integrated DNA Technologies, Coralville, IA, USA), and universal transactivating crRNAs was prepared according to a previously described protocol (36). The transformation of *C. auris* was performed via electroporation using approximately 1 µg of each construct. Transformants were then selected at 30°C on YPD agar containing 100-mg/mL nourseothricin (Jena Bioscience GmbH, Germany) for constructs containing the NatR selection cassettes. PCR screening was used to confirm proper incorporation of the constructs into the transformants, while sequencing was employed to validate the presence of mutations.

### Flow cytometric measurement of efflux using Nile red

To evaluate efflux in strains exhibiting increased manogepix MIC levels, intracellular concentrations of the fluorescent dye Nile red were measured using flow cytometry (37). *C. auris* cells (2.0 × 10^7^ cells/mL) were precultured in YPD medium at 30℃ for 4 h. Nile red was added at a final concentration of 7 µM. After incubation in the dark for 20 min with agitation at 30°C, 1 mL of the subculture was centrifuged, pelleted, and dissolved in 1 mL of 10-fold diluted PBS. The suspension was immediately used for flow cytometric analysis. Fluorescence from 20,000 cells was analyzed using a BD LSRFortessa X-20 (BD Biosciences, San Jose, CA, USA).

### Real-time qRT-PCR

Real-time qRT-PCR was performed as previously described (38). In brief, total RNA was extracted from logarithmic-phase cells grown in YPD broth using the RNeasy Plus Mini Kit (Qiagen, Valencia, CA, USA) and then reverse-transcribed into first-strand cDNA using the QuantiTect Reverse Transcription Kit (Qiagen). Three microliters of the resulting cDNA were used as the template for individual PCR using the QuantiTect SYBR Green PCR Kit (Qiagen) and gene-specific primers. qRT-PCR was performed in a 96-well plate using a QuantStudio 12 K Flex System (Thermo Fisher Scientific). The mRNA abundance of the target genes was normalized to that of *ACT1*. RNA extraction and qRT-PCR were performed in triplicate in three independent experiments.

### Statistical analysis

All statistical analyses were performed using Prism software, version 9.5.1 (GraphPad Software, Inc., La Jolla, CA, USA). Differences between groups were determined by two-tailed Student’s *t*-test. A *P* < 0.05 was considered significant.

## Data Availability

The data supporting the findings of this study are available in the paper and its supplemental material.

## SUPPLEMENTAL MATERIAL

Tables S1 and S2 aac.01508-24-s0001.docx MICs and primer list.

Figures S1 and S2 aac.01508-24-s0002.pdf Flow cytometric measurement and diagrams of genome editing.

ASM does not own the copyrights to Supplemental Material that may be linked to, or accessed through, an article. The authors have granted ASM a non-exclusive, world-wide license to publish the Supplemental Material files. Please contact the corresponding author directly for reuse.
