# Supplementary material for: TAC1b mutation in Candida auris decreases manogepix susceptibility owing to increased CDR1 expression
Source: Antimicrob Agents Chemother. 2024 Dec 18;69(2):e01508-24. doi: 10.1128/aac.01508-24 (PMC11823642; doi:10.1128/aac.01508-24)
Supplement: Tables S1 and S2 — MICs and primer list. [file aac.01508-24-s0001.docx]

**Supplementary Table S1. The MICs of 100 isolates from YPD agar containing manogepix**

| **Manogepix MIC (μg/ml)** | **0.03** | **0.06** | **0.125** | **0.25** | **0.5** |
| --- | --- | --- | --- | --- | --- |
| **Number of isolates** | **6** | **12** | **46** | **32** | **4** |

**Supplementary Table S2. The primers used in this study**

| For gene sequencing | | |
| --- | --- | --- |
| Aim | Primer | Sequence (5’-3’) |
| *GWT1* sequencing | GWT1-F | TCGTAGTAACCTTCGGACC |
|  | GWT1-R | GCACTCGTTTTCTGATGTC |
|  | GWT1-F-74 | CGTAACCCTCTTTTGCTATGG |
|  | GWT1-F600 | GCTTCTACACAACACCCTG |
|  | GWT1-F1108 | GCAAGGGAATCTTTTCACAC |
| *TAC1a* sequencing | TAC1a-F | CCATCTACTCCAATAAGACCAC |
|  | TAC1a-R | CTACCGTTTCTGAGCCTTC |
|  | TAC1a-F-119 | CCTAACGCCTACAATCCTC |
|  | TAC1a-F845 | AATGTCAGTTTTCCTCGG |
|  | TAC1a-F1672 | ACTTTTGAAGACGAGGACG |
|  | TAC1b-F | GAAGGCTCAGAAACGGTAG |
|  | TAC1b-R | GGAGAGAGTGTCTAAGGACC |
| *TAC1b* sequencing | TAC1b-F | GAAGGCTCAGAAACGGTAG |
|  | TAC1b-R | GGAGAGAGTGTCTAAGGACC |
|  | TAC1b-F-356 | ATTACGATGATGCGTTCCC |
|  | TAC1b-F379 | AGCAACAACAACGCTTCC |
|  | TAC1b-F1216 | GAACTTGGTCTTCACAGATTC |
|  | TAC1b-F1952 | CCATGATGAACTTGATTGC |
| *MRR1* sequencing | MRR1-F | CGCTCCACTCTTAGAAAAATGGTC |
|  | MRR1-R | TTGGTCATAAACTCAATATCCCTCC |
|  | MRR1-F-125 | TCCGAGATGTGTGAAATG |
|  | MRR1-F793 | CATCCAGACCTTGACATTC |
|  | MRR1-F1369 | AAGCATACTTTGTCCTTGGG |
|  | MRR1-F1929 | TCAGGTTCTAAACTCTGTGC |
|  | MRR1-F2174 | ATAGAGCCACTACCATCCG |
| *ZCF29* sequencing | ZCF29-F | GTTTTTGGAAGGAAGCAATC |
|  | ZCF29-R | GTGATTGACGGTGACTACG |
|  | ZCF29-F-325 | GAGAAGGTATTGACCGAGC |
|  | ZCF29-F650 | CAGATGAAGAACTGCGAG |
|  | ZCF29-F1385 | AGAATCTTAGGGAGACCGC |
| For introduction of *TAC1b*^D865N^ mutation | | |
| *TAC1b* mutation introduction | TAC1b_MT1 | GAGTTGAAGGAAGAGTTTCGTC |
|  | TAC1b_MT2 | TGACCCGGCGGGGACGAGGCAAGCTCATACTATTTACAAAGTTAAAGCCC |
|  | TAC1b_MT3 | GATACTAACGCCGCCATCCAGTGTCCTTACCACGATTTTAACGATGA |
|  | TAC1b_MT4 | AGGCTATTGCAAAGTACGACC |
|  | TAC1b_MT5 | GGGCTTTAACTTTGTAAATAGTATGAGCTTGCCTCGTCCCCGCCG |
|  | TAC1b_MT6 | TCATCATCGTTAAAATCGTGGTAAGGACACTGGATGGCGGCGTTAGTATC |
|  | TAC1b_MT7 | CGCTTTTACTTCGAGCCTG |
|  | TAC1b_MT8 | ﻿ATGGGAGCTTTACCAGGATATC |
|  | TAC1b_MTgRNA | CTAGCAGATGCAAAGTCAACGTTTTAGAGCTATGCT |
| For deletion of *TAC1b* | | |
| *TAC1b* deletion | TAC1b_DL1 | ACGCTGCTTATGTTCAAGG |
|  | TAC1b_DL2 | ﻿TGACCCGGCGGGGACGAGGCAAGCTAGCTTCTTGAGATTCGAATGAGCGA |
|  | TAC1b_DL3 | GATACTAACGCCGCCATCCAGTGTCCTTACCACGATTTTAACGATGA |
|  | TAC1b_DL4 | AGGCTATTGCAAAGTACGACC |
|  | TAC1b_DL5 | ﻿TCGCTCATTCGAATCTCAAGAAGCTAGCTTGCCTCGTCCCCGCCG |
|  | TAC1b_DL6 | TCATCATCGTTAAAATCGTGGTAAGGACACTGGATGGCGGCGTTAGTATC |
|  | TAC1b_DL7 | ATCTACGAAAGAAGCACGG |
|  | TAC1b_DL8 | ﻿ATGGGAGCTTTACCAGGATATC |
|  | TAC1b_DLgRNA1 | ACCACGCAAGTCTCGAACAAGTTTTAGAGCTATGCT |
|  | TAC1b_DLgRNA2 | GTCAACAGGATGAAGTATGTGTTTTAGAGCTATGCT |
| For deletion of *CDR1* | | |
| *CDR1* deletion | CDR1_DL1 | CGAAGACAGCCAACACAAC |
|  | CDR1_DL2 | ﻿TGACCCGGCGGGGACGAGGCAAGCT﻿GGAGATGGAAAAGTGAAGATTTG |
|  | CDR1_DL3 | GATACTAACGCCGCCATCCAGTGTC﻿GTGCATACATTAATACGATAAGTCG |
|  | CDR1_DL4 | ﻿GACCAAAAGTCGCCCTTG |
|  | CDR1_DL5 | ﻿GGCAAATCTTCACTTTTCCATCTCCAGCTTGCCTCGTCCCCGCCG |
|  | CDR1_DL6 | ﻿CGACTTATCGTATTAATGTATGCACGACACTGGATGGCGGCGTTAGTATC |
|  | CDR1_DL7 | ACTGTATGGAGTGTAGCGAC |
|  | CDR1_DL8 | ﻿GATACGCTGTTTCTGTCTCTC |
|  | CDR1_DLgRNA1 | AATTCCATCAATGAGTATACGTTTTAGAGCTATGCT |
|  | CDR1_DLgRNA2 | TCGGTACCATATTTTTCTACGTTTTAGAGCTATGCT |
| For qRT-PCR | | |
| *ACT1* | ACT1-F | GAAGGAGATCACTGCTTTAGCC |
|  | ACT1-R | GAGCCACCAATCCACACAG |
| *CDR1* | CDR1-F | GAAATCTTGCACTTCCAGCCC |
|  | CDR1-R | CATCAAGCAAGTAGCCACCG |
| *CDR2* | CDR2-F | TATTGTCTATCGTGGCGTC |
|  | CDR2-R | GCTGGATGAGAGTATTGAAGAC |
| *MDR1* | MDR1-F | GAAGTATGATGGCGGGTG |
|  | MDR1-R | CCCAAGAGAGACGAGCCC |
