# Supplementary material for: TAC1b mutation in Candida auris decreases manogepix susceptibility owing to increased CDR1 expression
Source: Antimicrob Agents Chemother. 2024 Dec 18;69(2):e01508-24. doi: 10.1128/aac.01508-24 (PMC11823642; doi:10.1128/aac.01508-24)
Supplement: Figures S1 and S2 — Flow cytometric measurement and diagrams of genome editing. [file aac.01508-24-s0002.pdf]

Figure S1

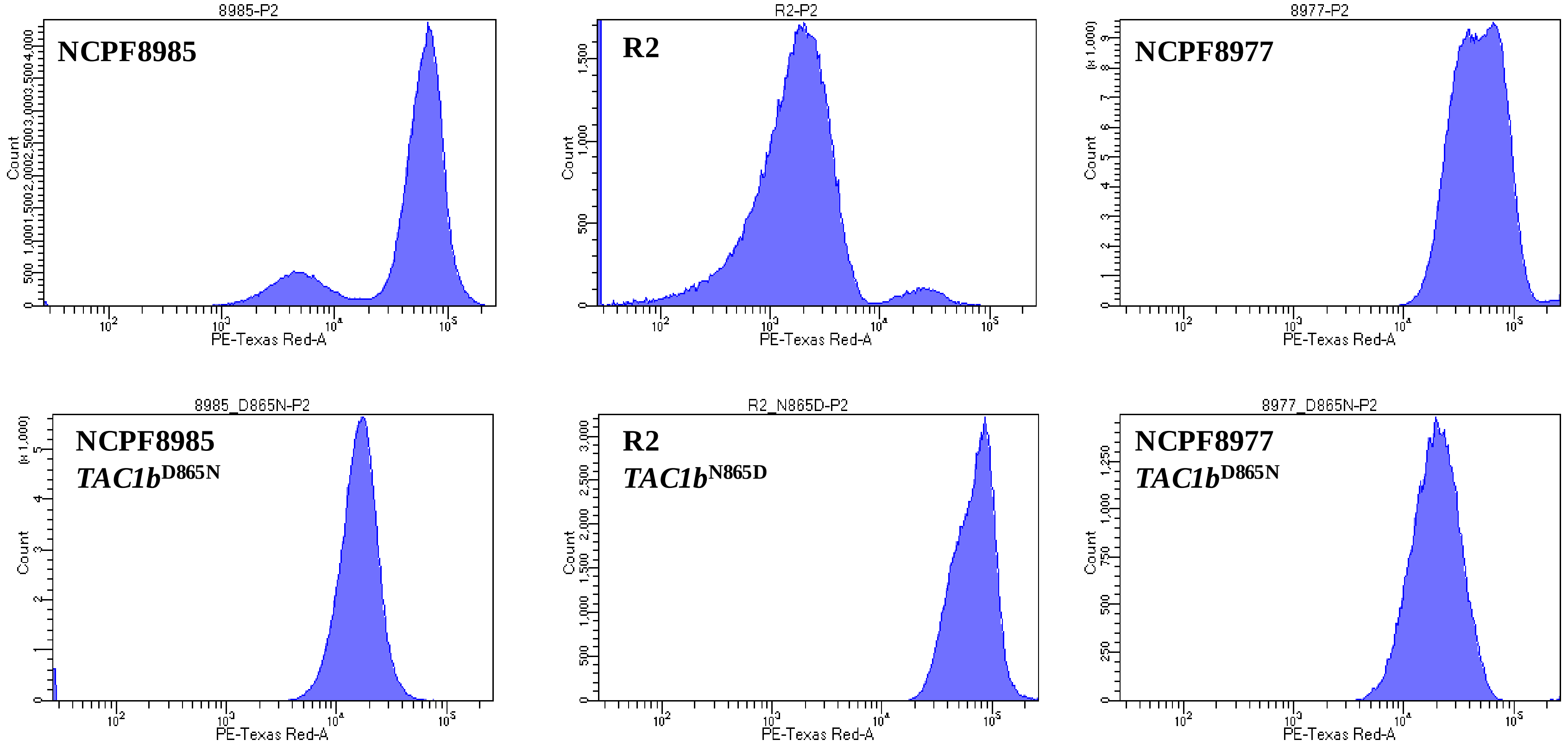


**Supplementary Figure S1. Flow cytometric measurement of efflux using Nile red**

Intracellular concentration of the fluorescent dye Nile red, a substrate of the efflux pump, was measured through flow cytometry in the wild-type and mutant strains of *TAC1b*. The fluorescence intensity of Nile red is shown on the X-axis, and the number of cells counted is shown on the Y-axis

Figure S2A

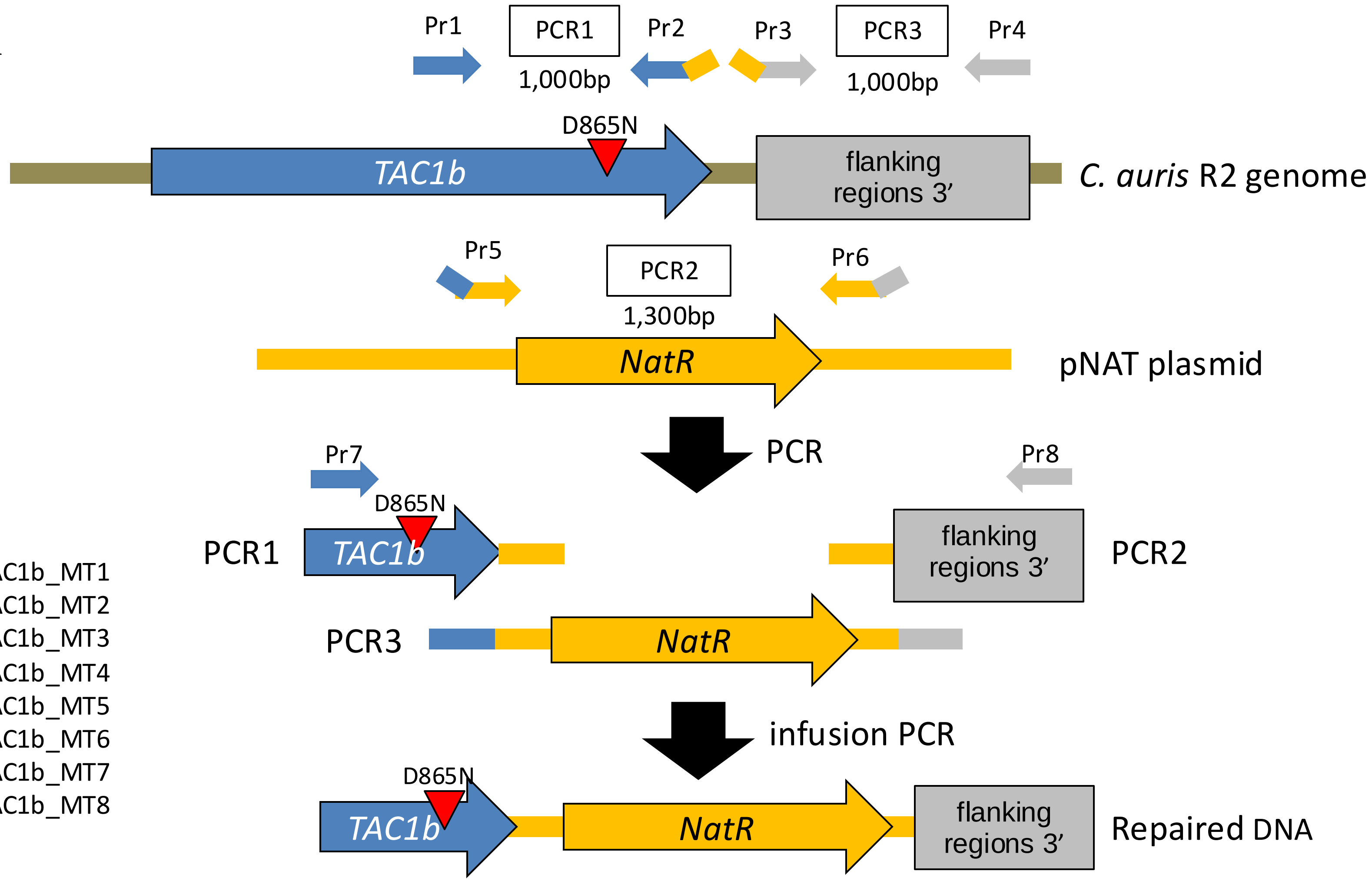


Pr1: TAC1b_MT1
Pr2: TAC1b_MT2
Pr3: TAC1b_MT3
Pr4: TAC1b_MT4
Pr5: TAC1b_MT5
Pr6: TAC1b_MT6
Pr7: TAC1b_MT7
Pr8: TAC1b_MT8

**Supplementary Figure S2A. Preparation of construct to generate *TAC1b*$^{D865N}$-mutant strain.** The repair cassette containing the nourseothricin-resistant (*NatR*) marker was obtained through fusion PCR using the following three overlapping DNA fragments: an approximately 1.0 kb DNA fragment from the genomic DNA of strain R2 containing the nucleotide change G2593A (corresponding to the D865N amino-acid change) of *TAC1b*; an approximately 1.3 kb DNA fragment containing the *NatR* marker from the plasmid pNAT; and an approximately 1.0 kb DNA fragment downstream of *TAC1b*. Infusion-PCR was performed using primers designed to overlap as shown in figure.

Figure S2B

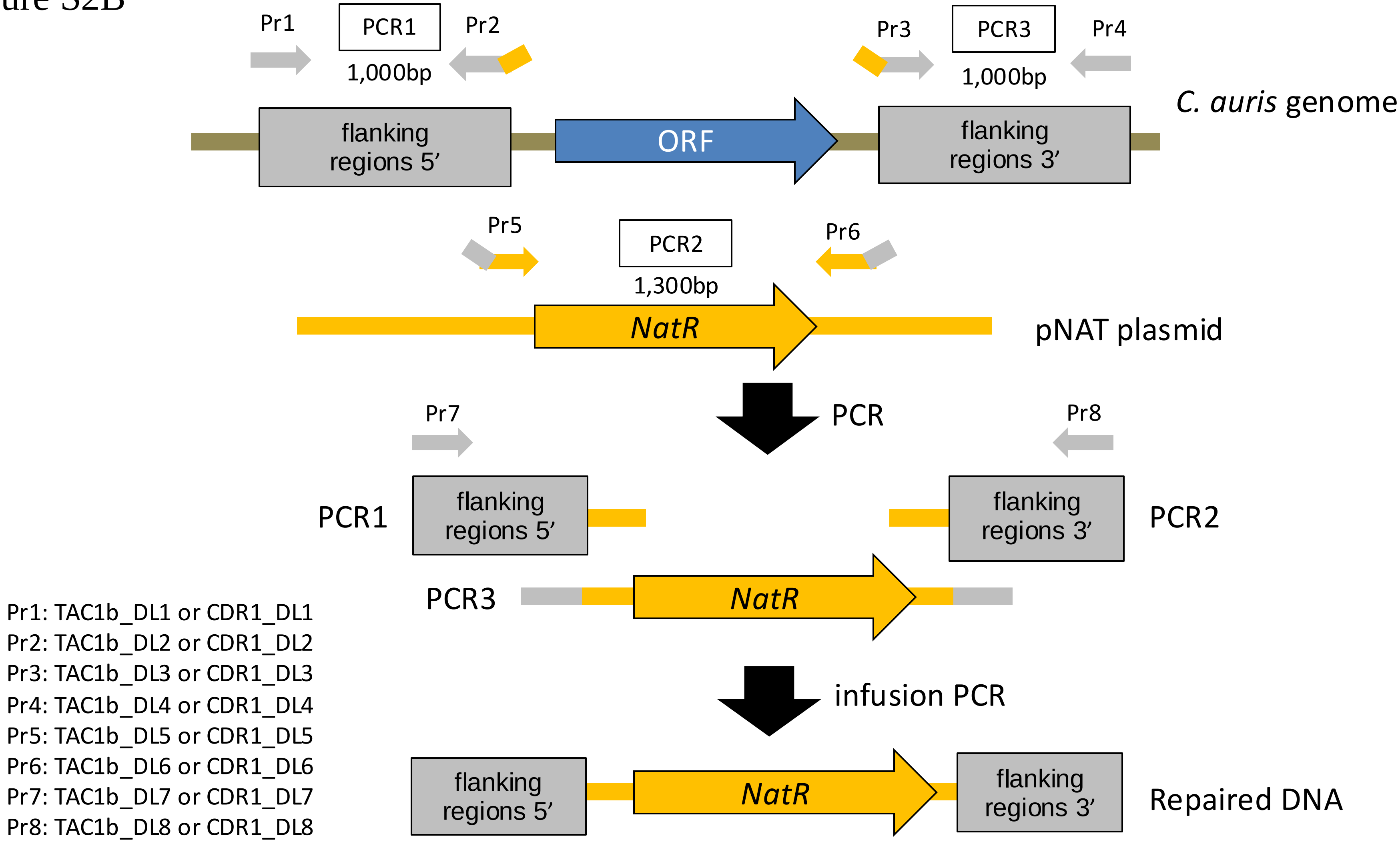


**Supplementary Figure S2B. Preparation of constructs to delete *TAC1b* and *CDR1*.**

The repair cassette contained the *NatR* marker and an approximately 1.0 kb fragment upstream and downstream of the open reading frame to be deleted. Infusion-PCR was performed using overlapping primers, as shown in the figure.

Figure S2C

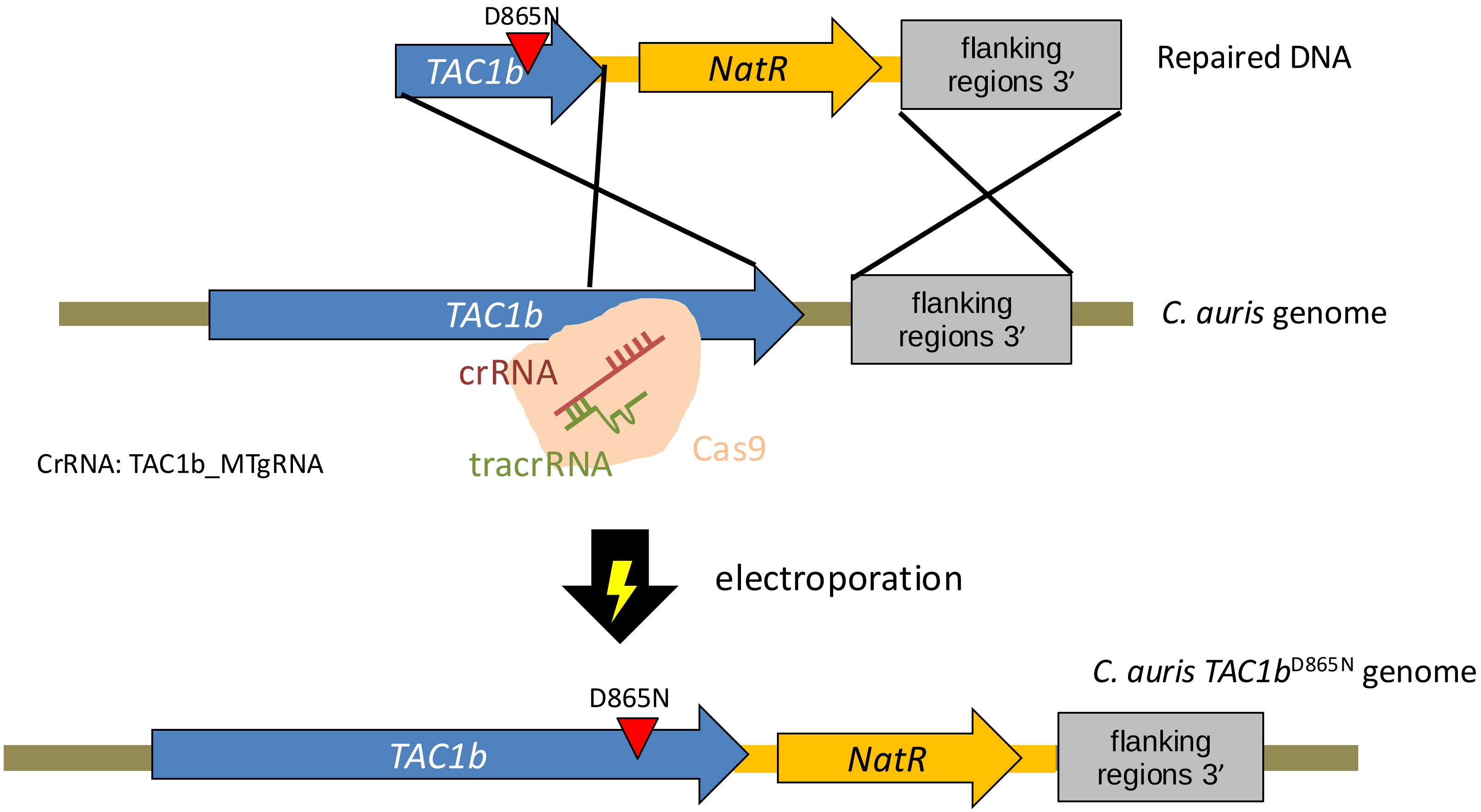


**Supplementary Figure S2C. Introduction of *TAC1b*$^{D865N}$ using CRISPR-Cas9.** The crRNA was designed to be close to the *TAC1b* mutation D865N. RNA-Cas9 protein complexes (RNPs) were formed by mixing crRNA, tracrRNA, and Cas9 endonuclease. RNPs and repaired DNA constructs were introduced into the cells through electroporation. As a result, a mutant strain was created in which D865N was introduced into *TAC1b* and *NatR* was inserted. N865D was introduced into the R2 strain as described above.

Figure S2D

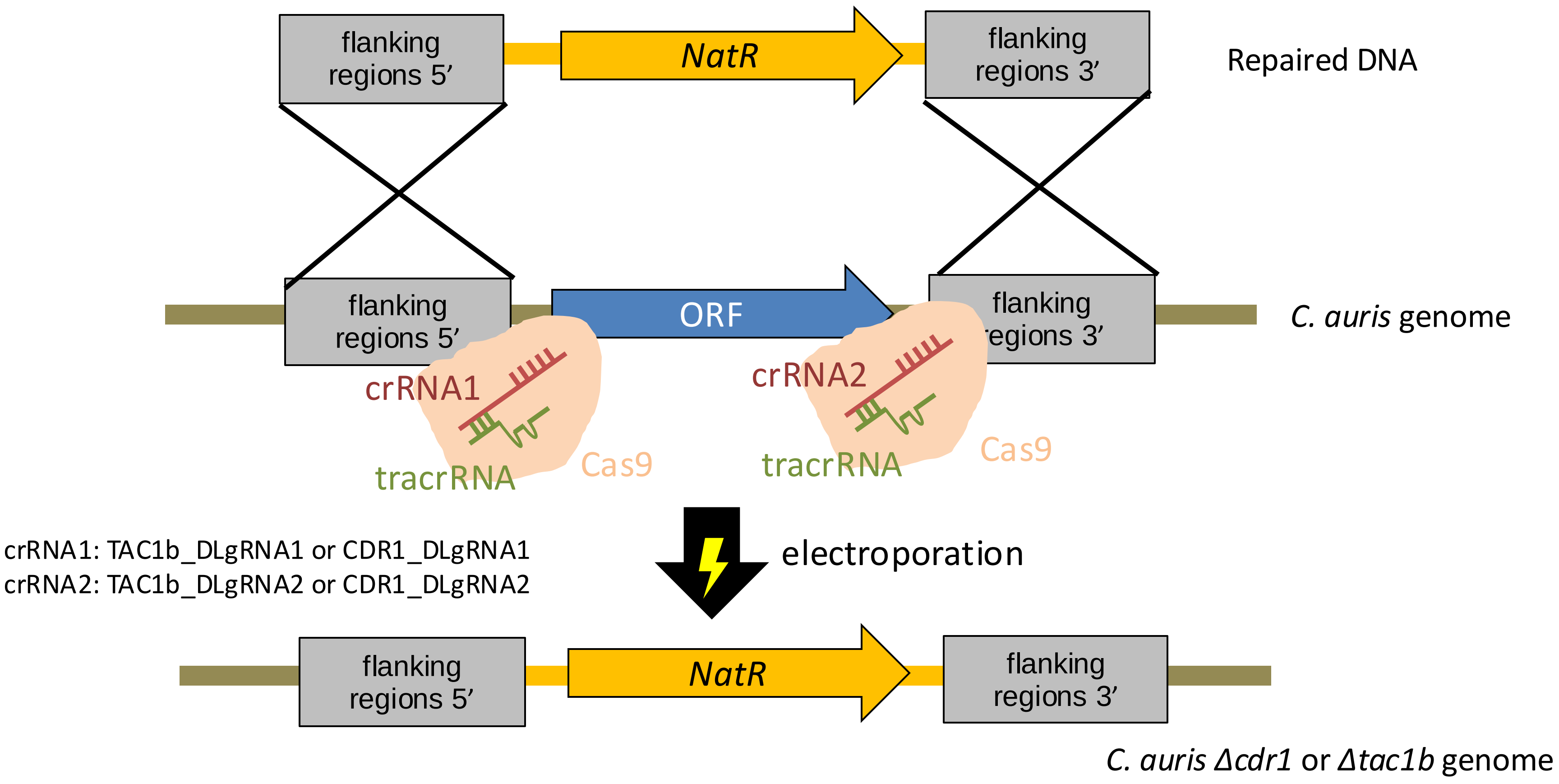


**Supplementary Figure S2D. Deletion of *TAC1b* and *CDR1* using CRISPR-Cas9.**
The crRNA was designed to induce Cas9 in the upstream and downstream regions of the ORF to be deleted. RNA-Cas9 protein complexes (RNPs) were formed by mixing crRNA, tracrRNA, and Cas9 endonuclease. RNPs and repaired DNA constructs were introduced into the cells by electroporation. As a result, gene deletion strains were created in which *TAC1b* or *CDR1* were replaced by *NatR*.
